# Interobserver variability of ventilatory anaerobic threshold in asymptomatic volunteers

**DOI:** 10.1186/s40248-019-0183-6

**Published:** 2019-06-10

**Authors:** Sabine Kaczmarek, Dirk Habedank, Anne Obst, Marcus Dörr, Henry Völzke, Sven Gläser, Ralf Ewert

**Affiliations:** 1 German Centre for Cardiovascular Research, Site Greifswald, Germany; 2 DRK Kliniken Berlin Köpenick, Klinik für Kardiologie, S.-Allende-Str. 2-8, 12555 Berlin, Germany; 3 Department of Internal Medicine B, University Hospital Greifswald, 17475 Greifswald, Germany; 4 Institute for Community Medicine, University Hospital Greifswald, 17475 Greifswald, Germany; 5 Department of Internal Medicine, Vivantes Klinikum Berlin-Spandau, 13585 Berlin, Germany

**Keywords:** Cardiopulmonary exercise testing, Anaerobic threshold, Observational study, Reproducibility

## Abstract

**Background:**

The ventilatory anaerobic threshold (VO_2_@AT) has been used in preoperative risk assessment and rehabilitation for many years. Our aim was to determine the interobserver variability of AT using cardiopulmonary exercise (CPET) data from a large epidemiological study (SHIP, Study of Health in Pomerania).

**Methods:**

VO_2_@AT was determined from CPET of 1,079 cross-sectional volunteers, according to American Heart Association guidelines. VO_2_@AT determinations were compared between two experienced physicians, between physicians and qualified medical assistants, and between physicians or medical assistants and software-based algorithms. For the first 522 data sets, the two physicians discussed discrepant readings to reach consensus; the remaining data sets were analyzed without consensus discussion.

**Results:**

VO_2_@AT was detectable in 1,056 data sets. The physicians recorded identical VO_2_@AT values in 319 out of 522 cases before consensus discussion (61.1%; intraclass correlation coefficient [ICC]: 0.90; 95% confidence interval [CI]: 0.88–0.92) and in 700 out of 1,056 cases overall (66.3%; ICC: 0.95; 95% CI: 0.95–0.96), with an interobserver difference of 0 ± 8% (95% limits of agreement [LOA]: ±161 mL/min). The interobserver difference was − 2 ± 18% (95% LOA: ±418 mL/min) between a physician and medical assistants, and − 19 ± 24% to − 22 ± 26% (95% LOAs: ±719–806 mL/min) between physicians or medical assistants and software-based algorithms.

**Conclusions:**

Experienced physicians show high agreement when determining AT in asymptomatic volunteers. However, agreement between physicians and qualified medical assistants is lower, and there is substantial deviation in AT determination between physicians or medical assistants and software-based algorithms. This must be considered when using AT as a decision tool.

## Background

Cardiopulmonary exercise testing (CPET) is a key method in clinical diagnostics, in assessment of illness severity, in determination and monitoring of therapy, and in prognostic stratification. The performance of CPET and interpretation of the results are generally well standardized internationally [1, 2]. Among other CPET parameters, the ventilatory anaerobic threshold (AT) has been used in pre-operative risk assessment for many years [3], and recently the predictive value of AT determination has expanded from valvular and thoracic surgery to pancreatic and liver resection [4]. An increase of AT is a decisive response to exercise prescription in patients with chronic heart failure [5, 6], pulmonary disease [6] or stroke [7]. The importance of AT and its exact determination is acknowledged in recent guideline updates [8, 9].

AT is defined as the exercise level at which ventilation (VE) begins to increase exponentially relative to the increase in oxygen uptake (VO_2_) [10]. There are wide differences in AT detection procedures and terminology [11, 12]. From a strict physiological viewpoint, there are two ventilatory thresholds, the first reflecting the transition from aerobic to anaerobic metabolism, and the second one from anaerobic metabolism to metabolic acidosis. Throughout this paper, we use the term ‘AT’ to refer to the first threshold. Methodological basics and practical guidelines for AT determination have been thoroughly reviewed elsewhere [9, 13].

The American Heart Association (AHA) Scientific Statement on CPET in adults [10] notes that confidence in determining AT may be increased by having two or three independent, experienced observers perform the calculation. If AT is calculated by software-based algorithms, it should be checked by an individual experienced in CPET and its assessment [10]. The repeatability of CPET parameters in healthy individuals [14] and intraindividual determination of the AT (test-retest comparison) in chronic disease cohorts [15, 16] is generally high. However, studies of interobserver variability of AT are limited. They refer to different populations, including healthy volunteers [17] and patients with heart failure [16, 18–20], congenital heart failure [21], pulmonary arterial hypertension [22], chronic obstructive pulmonary disease (COPD) [23] and mixed etiologies [24, 25]. Furthermore, the published studies vary substantially in terms of sample size (from *n* = 6 to *n* = 428) and statistical interpretation. Studies comparing software-based AT determination with visual determination of AT by clinically experienced readers have produced heterogeneous results [23, 25].

Therefore, the aim of our study was to determine the interobserver variability of AT using CPET data from a large population-based epidemiological study (Study of Health in Pomerania [SHIP]).

## Methods

### Study design and participants

SHIP is a large epidemiological study of 4,308 volunteers (age 20–79 years) drawn from the citizens registry of northeast Germany (West Pomerania). The volunteers were first evaluated from 1997 to 2001 (SHIP-0). The third follow up study (SHIP-3) re-evaluated 1,738 volunteers from the initial sample between 2014 and 2016. The methodological details of the overall study [26] and its pneumological aspects [27] have been published previously.

SHIP-3 was completed by 1,718 volunteers; 1,128 (65.6%) of the volunteers underwent CPET, and 1,079 had full data available and were included in the analyses presented here. All anamnestic data based on survey of the volunteers by professional interviewer and covered smoking status (current, former, never smoker), physical activity, previous myocardial infarction, atrial fibrillation, heart failure, heart operation, pacemaker, chronic bronchitis, and asthma. These demographic data are presented in Table 1.

**Table 1:** Descriptive Statistics of the Study Population (*N* = 1,056)

Parameter	Missing data, n	*n* (%)	Median (interquartile range)
Men		515 (48.8%)	
Age, years			60 (49–69)
Weight, kg			79 (69–90)
Height, cm			169 (162–176)
BMI, kg/m^2^			27.3 (24.6–30.6)
BMI ≥ 30 kg/m^2^		298 (28.2%)	
Smoking status	2		
Never smokers		404 (38.3%)	
Former smokers		485 (46.0%)	
Current smokers		165 (15.7%)	
Physically active^a^		870 (82.4%)	
Myocardial infarction	2	25 (2.4%)	
Atrial fibrillation	8	54 (5.1%)	
Heart failure	125	30 (3.2%)	
Fractional shortening below normal^b^	324	2 (0.3%)	
Heart operation	1	22 (2.1%)	
Pacemaker	3	5 (0.5%)	
Previous pulmonary disease	2	52 (4.9%)	
Chronic bronchitis^c^	1	53 (5.0%)	
Asthma	4	55 (5.2%)	
Maximum exercise duration, s			536 (420–670)
Maximum power, W			148 (132–196)
peak VO_2_, mL/min	2		1,812 (1,482–2,292)
VO_2_@AT, mL/min			1,009 (867–1,204)
VO_2_@AT/peak VO_2 reference_, % ^d^			54.6 (47.1–63.7)
VO_2_@AT/peak VO_2 reference_ < 45% ^d^		163 (15.4%)	
RER@AT			0.82 (0.77–0.86)
FEV_1_/FVC, %	3		75.7 (71.4–79.7)
FEV_1_/FVC < 70%		199 (18.9%)	

Anamnestic data based on survey by professional interviewer. Continuous data are expressed as the median (25^th^; 75^th^ quartile). Nominal data are given as percentages

^a^ Volunteers were asked about their physical activity and categorized as “Physically active” in case of 1–2 h of activity per week in summer *and* winter

^b^ The echocardiographic parameter “fractional shortening” was calculated as (left ventricular diastolic – systolic diameter [in cm]) × 100. Pathologic values were < 19% in males and < 21% in females

^c^ Subgroups may overlap

^d^ Predicted values were calculated according to Gläser S et al. [28] These were: peak VO_2_ in males = 254.76–22.69 × age [years] + 17.25 × height [cm] + 4.41 × weight [kg]; and peak VO_2_ in females = −54.74 – 9.81 × age [years] + 9.92 × height [cm] + 8.06 × weight [kg]

*BMI* Body mass index, *peak VO*_*2*_ Peak oxygen uptake, *VO*_*2*_*@AT* Oxygen uptake at the aerobic-anaerobic threshold, *RER* Respiratory exchange ratio, *FEV*_*1*_*/FVC* Forced expiratory volume in 1 s/forced vital capacity

### CPET

All volunteers underwent symptom-limited exercise testing until maximum exhaustion on an electromagnetically braked bicycle ergometer in an upright sitting position (Ergoselect 100, Ergoline, Germany), using the modified Jones protocol: 3 min measurements at rest, 1 min unloaded cycling, stepwise increase of workload by 16 W/min, and 5 min recovery. Gas exchange and ventilation were measured breath by breath using an Oxycon Pro® system (VIASYS Healthcare GmbH, Hoechberg, Germany) with a CPET (7450 V2) mask. Calibration was performed before every exercise test [28].

### Calculation of AT

Values of AT are given as VO_2_ at the aerobic-anaerobic threshold (VO_2_@AT) in mL/min. AT was determined manually according to current guidelines [9, 10, 13]. First, the slope of the VCO_2_ versus VO_2_ relationship was analyzed, and AT identified as the point of transition in the VCO_2_ versus VO_2_ slope from < 1 to > 1 (“V-slope method”). Second, in cases where the V-slope method could not be applied, AT was defined as the lowest point of the ventilatory equivalent for oxygen (VE/VO_2_). The software-based AT determination used the VIASYS software calculation tool (JLab Labmanager V5.32.0). Manual assessment included data from the fourth minute of exercise until a respiratory exchange ratio (RER) of 1 was exceeded. Raw data were averaged at intervals of 10 s for both methods (manual and software-based), and the AT was determined using 30-s rolling averages calculated every 10 s.

### Interobserver comparisons

Two physicians and two medical technical assistants determined values of AT manually. The physicians had 2 years and 20 years of experience in CPET, and both underwent a training phase in which they evaluated the same 400 CPET data sets (using 10- and 30-s intervals) to ensure adherence to the AHA guidelines [10] for determination of AT (data not shown). The medical technical assistants completed a special education program and were certified in the performance, supervision, and interpretation of CPET.

**Fig. 1:**
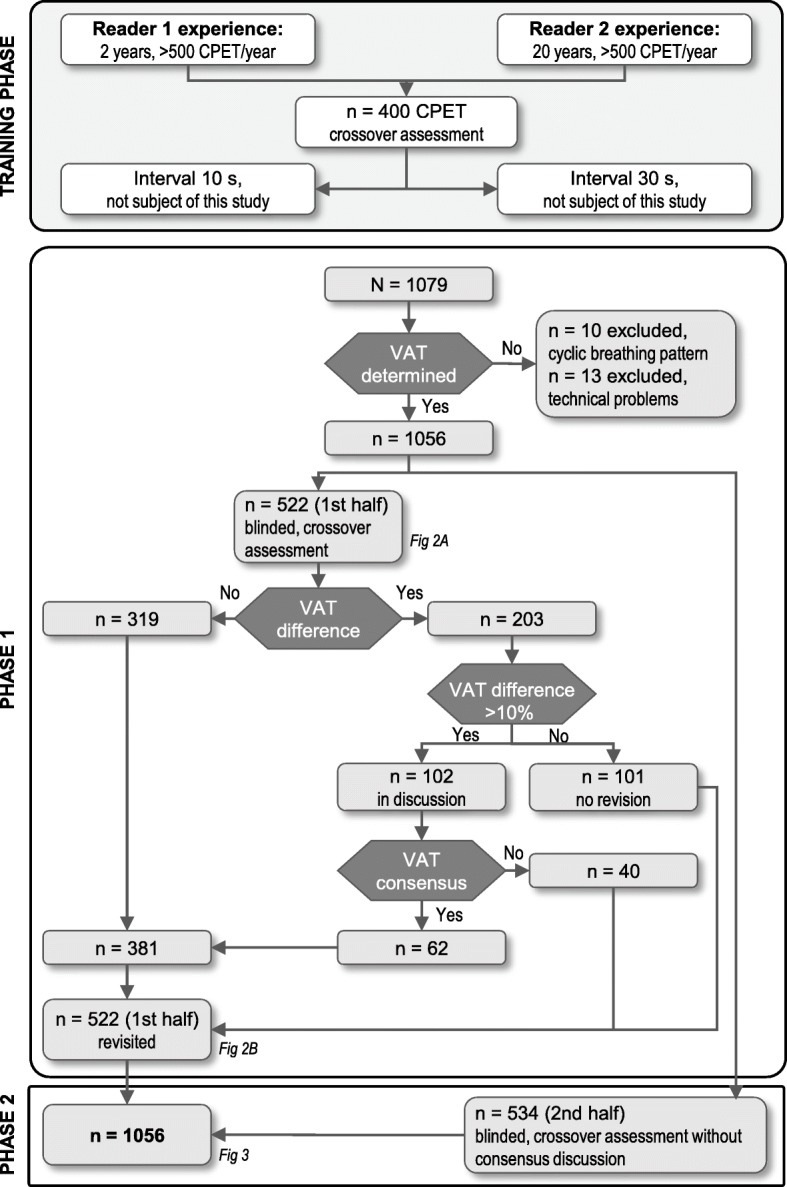
Flow chart summarizing the study of interobserver agreement in AT assessment between two physicians. CPET = cardiopulmonary exercise training; AT = ventilatory anaerobic threshold

Software-based AT values and values determined by medical assistants were used directly in the interobserver agreement analysis. The determination of AT values by the physicians was conducted in two phases (Fig. 1). In phase 1 (the first 522 CPETs), each physician independently determined the AT for each CPET, and cases with a difference of > 10% underwent consensus discussion between the two physicians to reach agreement (if agreement was not reached, the differing values as originally calculated were taken into the statistical analysis). In phase 2, the remaining 534 CPETs were analyzed independently by each physician without consensus discussion.

Levels of interobserver agreement were calculated between the two physicians in phase 1 (before and after the consensus discussion) and in phases 1 and 2 combined (after the phase 1 consensus discussion). AT values determined by the medical assistants were compared with the values determined by each of the physicians (after the consensus discussion). Finally, AT values determined by the medical assistants and each of the physicians (after the consensus discussion) were compared with those determined by the software.

### Statistical analysis

Continuous data are expressed as median (interquartile range), and nominal data are given as percentages.

To assess interobserver agreement, the intraclass correlation coefficient (ICC) was calculated. Bland-Altman plots were used to compare the VO_2_@AT calculations of the different readers and the software. In addition, a Passing-Bablok regression analysis was performed comparing software- and manually-derived VO_2_@AT.

The reliability of VO_2_@AT readings was assessed using the typical error (TE), the coefficient of variation of the TE (CV_TE_), and limits of agreement, expressed as absolute values (LOA) and as a percentage of the mean VO_2_@AT (LOA%).

Statistical analyses were performed using SAS version 9.4 (SAS Institute, Inc., NC, USA).

## Results

Out of the 1,079 available data sets, 10 (0.9%; all showing an unstable cyclic breathing pattern) had non-detectable AT according to at least one physician reader and were therefore excluded from the analysis (7 [0.6%] were deemed uninterpretable by both physicians). Thirteen data sets with technical problems were also excluded. Thus, the final interobserver agreement analysis contained 1,056 data sets. The study participants represented a typical and asymptomatic but not strictly healthy population with a median body mass index of 27.3 kg/m^2^; 82.4% self-reported being physically active, 15.7% were active smokers and 5.0% had chronic bronchitis. In total, 18.9% had pulmonary obstruction (forced expiratory volume in 1 s/forced vital capacity < 70%). The median RER at AT was 0.82 (0.77–0.86), consistent with the reported training status and sure beyond exercise to exhaustion.

### Interobserver agreement between physicians

**Fig. 2:**
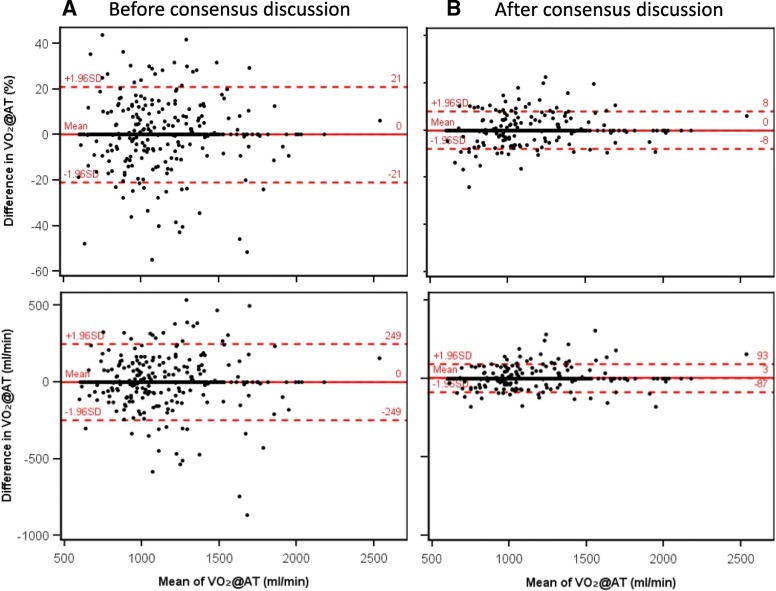
Bland-Altman plots of ventilatory anaerobic threshold (expressed as VO_2_@AT) determined by two physician readers in study phase 1, (**a**) before and (**b**) after consensus discussion (*n* = 522 data sets). Upper and lower plots show percentage difference and difference in mL/min, respectively. SD = standard deviation; VO_2_@AT = oxygen uptake at the anaerobic threshold

**Fig. 3:**
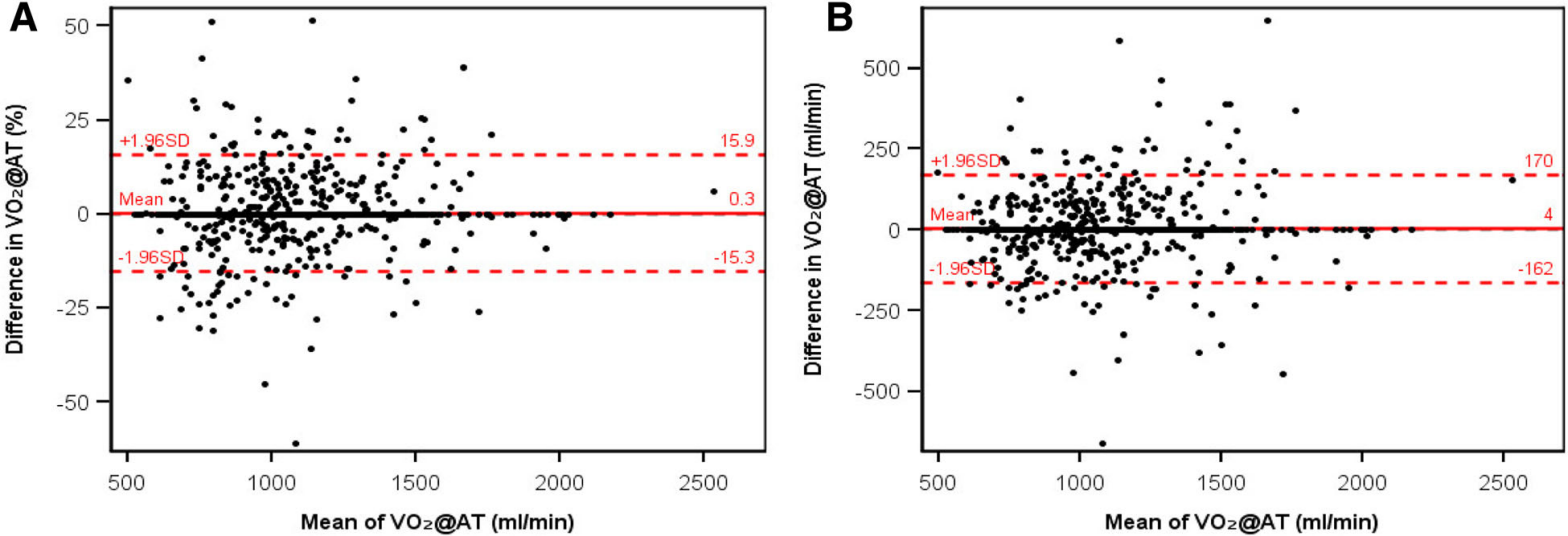
Bland-Altman plots of ventilatory anaerobic threshold (expressed as VO_2_@AT) determined by two physician readers in study phase 1 (after consensus discussion) and phase 2 combined (n = 1,056 data sets). **a** Percentage difference. **b** Difference in mL/min. SD = standard deviation; VO_2_@AT = oxygen uptake at the anaerobic threshold

Phase 1 showed complete agreement (i.e. VO_2_@AT difference = 0) between the two physicians in 319 of 522 cases (61.1%). The mean difference was − 0.1 mL/min and the LOA ±250 mL (Fig. 2 and Table 2). 103 cases showed a difference of > 10% in VO_2_@AT; following consensus discussion of these cases, the agreement increased to 382 of 522 cases (73.2%, Fig. 2 and Table 2). In phases 1 and 2 combined (after phase 1 consensus discussion), the physicians agreed in 700 out of 1,056 cases (66.3%), with an ICC of 0.95 (0.95–0.96) (Fig. 3 and Table 2). The mean difference was + 5 mL/min and the LOA ±161 mL/min, with an interobserver variability between physicians of ±8%.

**Table 2:** Interobserver Agreement for Determination of Ventilatory Anaerobic Threshold (VO_2_@AT)

	n	Mean VO_2_@AT (±SD), mL/min	Mean difference (±SD_d_), mL/min	95% LOA,^a^ mL/min	TE^b^	Mean difference (± SD_d_), %	95% LOA,^a^ %	ICC (95% CI)	CV_TE_, ^c^ %
Before consensus discussion									
Reader 1 vs reader 2 (phase 1)	522	1,093 (±279)	0 (±127)	±250	90	0 (±11)	±21	0.901 (0.884–0.916)	8.2
After consensus discussion									
Reader 1 vs reader 2 (phase 1)	522	1,085 (±283)	3 (±46)	±90	32	0 (±4)	±8	0.987 (0.985–0.989)	3.0
Reader 1 vs reader 2 (phase 1 + 2)	1,056	1,048 (±246)	5 (±82)	±161	58	0 (±8)	±15	0.952 (0.946–0.957)	5.6
Reader 1 vs trained assistants	794	1,073 (±292)	−36 (±213)	±418	151	−2 (±18)	±34	0.759(0.728–0.787)	14.0
Reader 2 vs trained assistants	793	1,070 (±289)	−42 (±208)	±408	147	−3 (±18)	±34	0.762 (0.731–0.790)	13.8
Reader 1 vs computer analysis	655	1,215 (±371)	−313 (±409)	±801	289	−22 (±26)	±51	0.350 (0.281–0.415)	23.8
Reader 2 vs computer analysis	658	1,210 (±367)	−321 (±411)	±806	291	−22 (±26)	±51	0.330 (0.260–0.396)	24.0
Trained assistants vs computer analysis	654	1,236 (±407)	−275 (±367)	±719	259	−19 (±24)	±48	0.519 (0.461–0.573)	21.0

^a^ 95% LOA = ±1.96 × SD_d_

^b^ TE = SD_d_ / √2

^c^ CV_TE_ = TE / mean VO_2_@AT × 100

*CI* Confidence interval, *CV*_*TE*_ Coefficient of variation of the TE, *d* Differences, *ICC* Intraclass correlation coefficient, *LOA* Limits of agreement, *SD* Standard deviation, *TE* Typical error, *VO*_*2*_*@AT* Oxygen uptake at the aerobic-anaerobic threshold [mL/min]

### Agreement between physicians and assistants

**Fig. 4:**
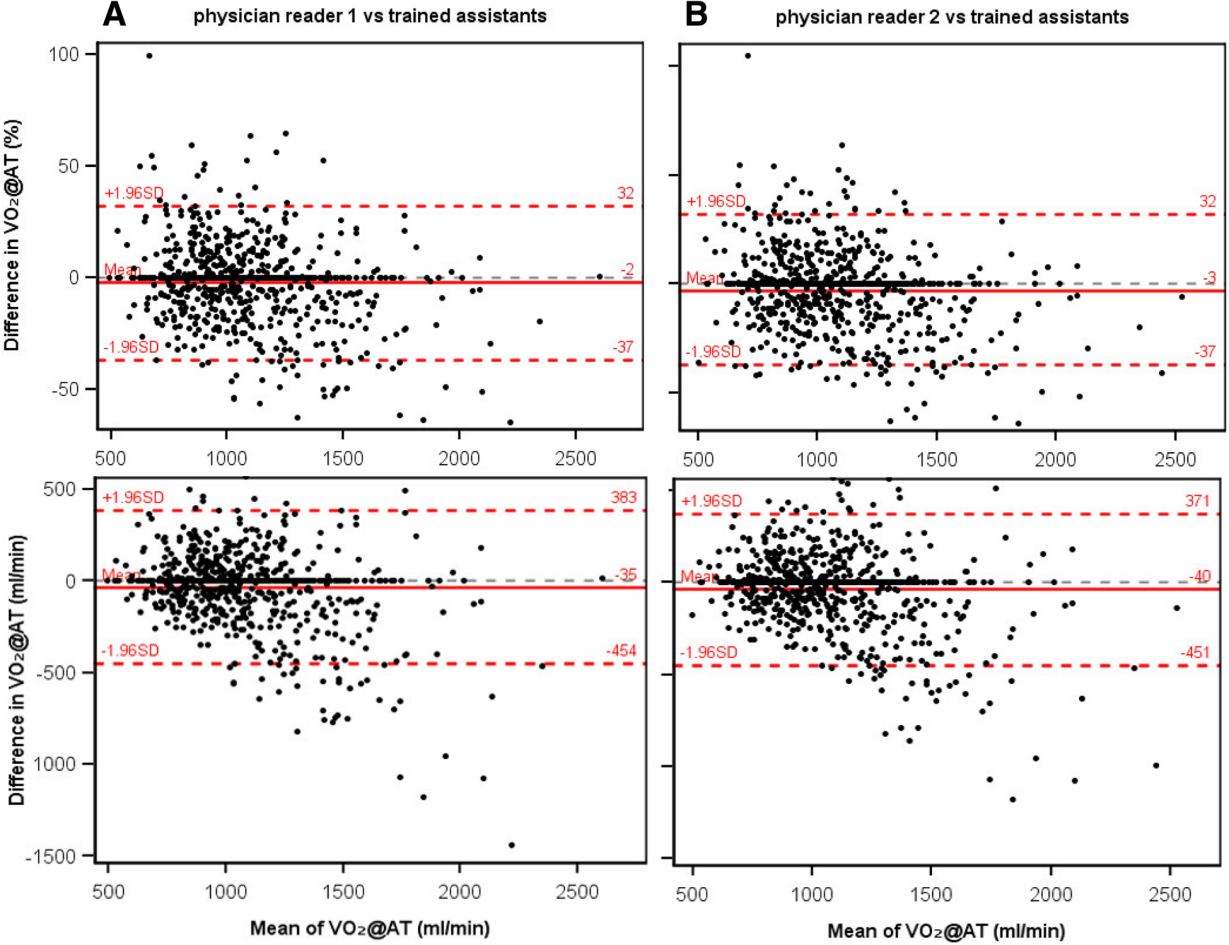
Bland-Altman plots of ventilatory anaerobic threshold (expressed as VO_2_@AT) determined by trained medical assistants compared with (**a**) physician reader 1 or (**b**) physician reader 2. Upper and lower plots show percentage difference and difference in mL/min, respectively. SD = standard deviation; VO_2_@AT = oxygen uptake at the anaerobic threshold

The interobserver agreement analysis between physician readers 1 and 2 and trained assistants included 794 and 793 data sets, respectively. There were no systematic differences in calculated VO_2_@AT between these groups (Fig. 4 and Table 2), although agreement was somewhat lower than that observed between the two physicians. The interobserver variability between physicians and medical assistants was ±18% (LOAs: ±408 and 418 mL/min).

### Manual versus software-based methods

**Fig. 5:**
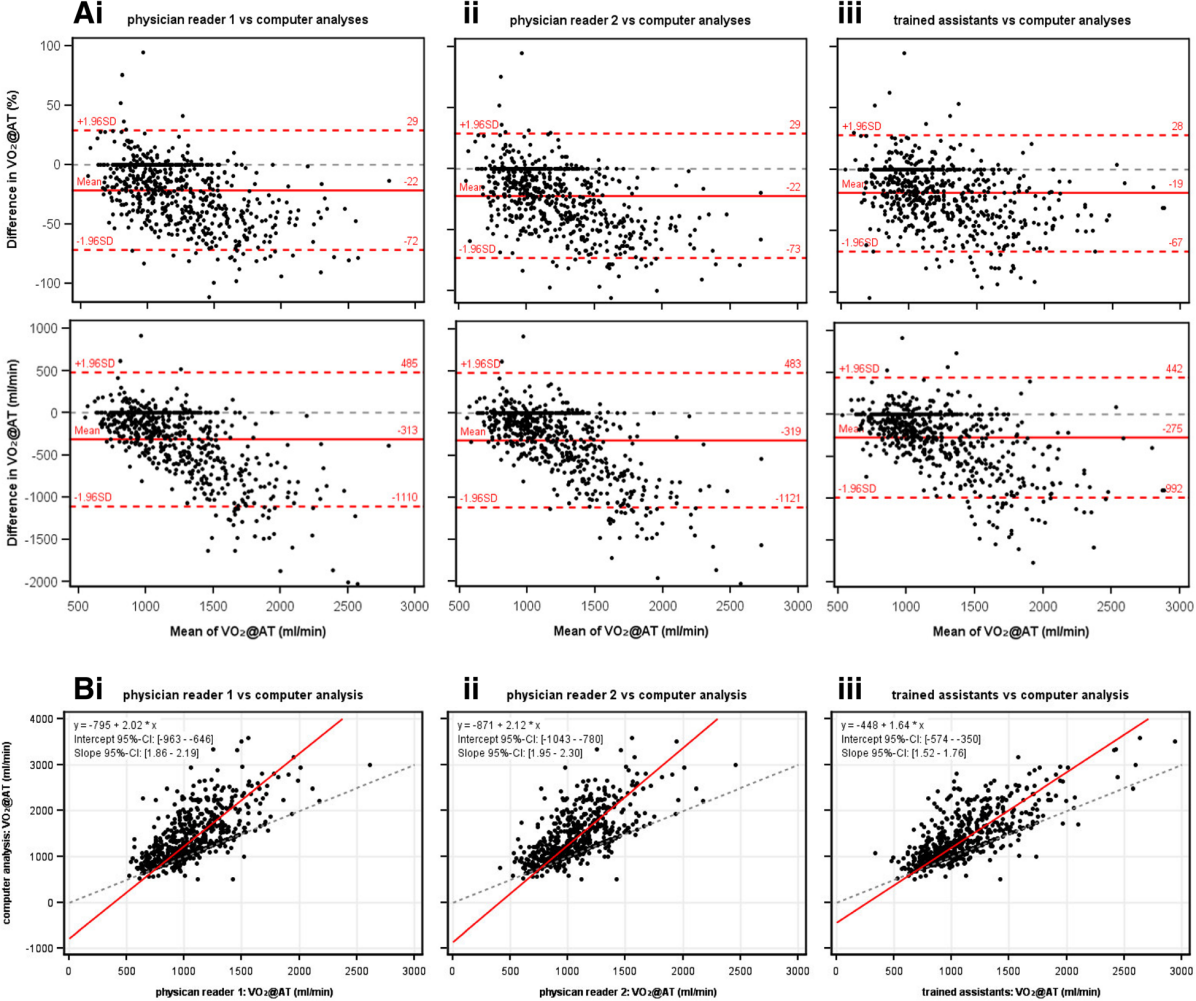
(**a**) Bland-Altman plots and (**b**) Passing-Bablok regression analysis of ventilatory anaerobic threshold (expressed as VO_2_@AT) determined manually by (i, ii) physician readers or (iii) trained assistants compared with VO_2_@AT derived from a software-based algorithm. Upper and lower Bland-Altman plots show percentage difference and difference in mL/min, respectively. CI = confidence interval; SD = standard deviation; VO_2_@AT = oxygen uptake at the anaerobic threshold

The comparison between software-based and manually-derived VO_2_@AT contained 655 (physician 1), 658 (physician 2), and 654 (medical assistants) data sets (Fig. 5 and Table 2). The software-based VO_2_@AT tended to be higher than the manually-derived VO_2_@AT at higher VO_2_@AT values (overall mean differences, 275–321 mL/min). The interobserver variability between the software and the physicians/assistants was ±24–26% (95% LOAs: ±719–806 mL/min).

## Discussion

Our epidemiological study contained over 1,000 participants, and experienced readers were able to determine an AT in > 99% of cases. This proportion is consistent with the literature: despite adequate exhaustion and experienced readers, AT determination is not always possible [10], especially in individuals with periodic breathing patterns (as seen in our study) and in patients with chronic heart failure (AT was indeterminate in 16% of 1,679 tests in the Fix-Heart Failure-5 trial [16], 17% of 398 tests in the Heart Failure-ACTION trial [15], and 29% of 331 tests in a multicenter trial by Cohen-Solal et al. [19]). In contrast to these studies, we intended to determine AT in a typical population, and the demographic characteristics of our study population matched this criterion.

Our data show that after a training period with 400 exercise tests, the subsequent analysis of > 1,000 data sets by two physicians resulted in an ICC of 0.95 (95% confidence interval [CI]: 0.95–0.96), and VO_2_@AT values within the 95% LOA had a difference of ±161 mL/min (SD_d_ ± 8%). In phase 1 (the first 522 data sets), the consensus discussion increased the ICC to 0.99 and lowered the difference within the 95% LOA to ±90 mL/min (SD_d_ ± 4%). A comparably high ICC of 0.98 (95% CI: 0.97–0.99) was shown in a study of 23 healthy volunteers undergoing cycle ergometer testing [23]. By contrast, analysis of 92 patients with COPD in the same study resulted in an ICC of 0.72 (95% CI: 0.60–0.81) [23]. Studies of AT in patient populations undergoing exercise testing with a treadmill ergometer revealed a range of ICCs, from 0.64 in 16 patients with chronic heart failure [20] to 0.85 in 445 tests of patients before vascular operation [25] and 0.88–0.97 (with three readers) in 13 children with congenital heart failure [21].

The software-based algorithm used in our study showed substantial differences in all statistical parameters when compared with manual assessment by physicians or medical assistants (Table 2). However, comparison with previously published studies is difficult, because the software-based AT determination was performed using different algorithms. Dubé et al. [23] used the LAB Manager version 5.3.0.4 (Cardinal Health, Höchberg, Germany) and Vainshelboim et al. [25] used the COSMED system (Rome, Italy) and special analytic software, whereas our study used VIASYS software (JLab Labmanager V5.32.0).

The absolute differences in the AT values calculated by the physician readers deserve special interest from the clinician’s viewpoint. Our study assessed AT in asymtomatic volunteers and showed a difference of 5 ± 82 mL/min (mean ± SD_d_) between the two readers, which corresponds to a 95% LOA of ±161 mL/min. The study of patients with COPD mentioned above [23] showed a mean interobserver difference of 189 ± 115 mL/min (95% LOA: − 35-413 mL/min). In patients with chronic heart failure the mean interobserver difference was 13 ± 105 mL/min (95% LOA: − 194-220 mL/min) [16]. Other authors reported median interobserver differences, expressed in relation to body weight (mL/kg/min) [18], in percent [24], or in absolute terms (mL/min) [22]. The latter study analyzed 42 patients with pulmonary hypertension and showed differences according to the readers’ experience, with median differences in AT ranging from 20 mL/min (for very experienced readers) to 60 mL/min (for less experienced readers). The median interobserver difference overall was 36 mL/min (6.4%) [22].

Although interobserver differences between physicians were low in our study, larger differences were seen when comparing medical assistants’ readings with those taken by physicians (95% LOA: approx. 400 mL/min) and particularly when comparing software-based versus manually-derived readings (95% LOA: ±719–806 mL/min). This is partly contradictory to the recent recommendations of the AHA which include AT determination in the pre-surgical risk assessment algorithm for non-cardiac surgery [8]. Possible consequences of our calculated 95% LOA can be shown on the basis of this recommendation: if the AT of a patient weighing 70 kg was determined by a physician to be 10.0 mL/kg/min, which is lower than the accepted cut-off for mortality risk in abdominal surgical procedures (11 mL/kg/min) [29], it could also be determined as 13.4 mL/kg/min (+ 34%, upper LOA) by a medical assistant or even 15.1 mL/kg/min (+ 51%) by a software-based algorithm. In this context, at least the software-based AT calculation could lead to a patient being classed (perhaps wrongly) as having an acceptable mortality risk. The determination of AT before exercise training in rehabilitation may be less critical; however, physicians should be aware of the LOA before prescribing aerobic exercise training. The benefit of these individual exercise doses has recently been shown in cardiac [30] and pulmonary diseases [6]. Exact determination of the AT and an awareness of the variability in AT calculation will enhance the application of AT in future pre-operative risk assessment, rehabilitation and study design.

### Limitations

The averaging of raw data was performed in accordance with the AHA Scientific Statement on CPET [10]: “for routine clinical use, if feasible, the averaging of data over 20- to 30-second intervals is generally sufficient to reduce the effect of random noise in breath-by-breath measurements”. Other guidelines [13] recommend a rolling averaging of data over 8–10 breaths for AT determination. Both the interval and the number of breaths can be delayed up to 15 breaths, leading to a smoothing of curves.

We primarily used the V-slope method to detect the AT. Future studies should take the average of different methods of AT calculation, because this could yield more accurate values than a single AT calculation, especially in healthy resp. asymptomatic volunteers [31].

In accordance with other authors, we excluded the first minute of exercise from the analysis. This should avoid confounding of our results by a “pseudo threshold”, which can be caused by hyperventilation at the start of exercise [23].

## Conclusions

In summary, our analysis of CPET data from > 1,000 asymptomatic volunteers shows varying degrees of interobserver variability and supports the need for independent assessment of essential CPET parameters by more than one reader. Furthermore, the data presented here may inform the calculation of statistical power in future clinical studies. Interobserver variability should be considered when determining AT values for pre-operative risk assessment and before prescription of aerobic exercise training.

## Abbreviations

AHA: American Heart Association
AT: Anaerobic threshold
BMI: Body mass index
CI: Confidence interval
COPD: Chronic obstructive pulmonary disease
CPET: Cardiopulmonary exercise testing
CV_TE_: Coefficient of variation of the typical error
FEV_1_: Forced expiratory volume in 1 s
FS: Fractional shortening
FVC: Forced vital capacity
ICC: Intraclass correlation coefficient
LOA: Limits of agreement
RER: Respiratory exchange ratio
SD: Standard deviation
SD_d_: Standard deviation of mean difference
SHIP: Study of Health in Pomerania
TE: Typical error
VE: Ventilation
VE/VO_2_: Ventilatory equivalent for oxygen
VO_2_: Oxygen uptake
